# Age modifies the association between pet ownership and cardiovascular disease

**DOI:** 10.3389/fvets.2023.1168629

**Published:** 2023-05-12

**Authors:** Katharine M. Watson, Ka Kahe, Timothy A. Shier, Ming Li

**Affiliations:** ^1^Department of Epidemiology and Biostatistics, Indiana University, Bloomington, IN, United States; ^2^Department of Obstetrics and Gynecology, Columbia University Irving Medical Center, New York, NY, United States; ^3^Department of Epidemiology, Columbia University Irving Medical Center, New York, NY, United States; ^4^Indiana Department of Natural Resources, Bloomington, IN, United States

**Keywords:** human animal bond, cardiovascular disease, pet ownership, one health, age, aging

## Abstract

**Introduction:**

Studies examining associations between pet ownership and cardiovascular disease have yielded inconsistent results. These discrepancies may be partially explained by variations in age and sex across study populations. Our study included 6,632 American Gut Project participants who are US residents ≥40 years.

**Methods:**

We first estimated the association of pet ownership with cardiovascular disease risk using multivariable-adjusted logistic regression, and further investigated effect modifications of age and sex.

**Results:**

Cat but not dog ownership was significantly associated with lower cardiovascular disease risk (OR: 0.56 [0.42, 0.73] and OR: 1.17 [0.88, 1.39], respectively). Cat and dog ownership significantly interacted with age but not sex, indicating that cardiovascular risk varies by the age-by-pet ownership combination. Compared to the reference group (40–64 years, no cat or dog), participants 40–64 years with only a cat had the lowest cardiovascular disease risk (OR: 0.40 [0.26, 0.61]). Those ≥65 years with no pets had the highest risk (OR: 3.85 [2.85, 5.24]).

**Discussion:**

This study supports the importance of pets in human cardiovascular health, suggesting optimal pet choice is age-dependent. Having both a cat and dog can be advantageous to people ≥65 years, while having only a cat may benefit those 40–64 years. Further studies are needed to assess causality.

## Introduction

Despite recent advances in medical and pharmaceutical treatments, cardiovascular disease (CVD) remains the leading cause of mortality in the US and worldwide (1, 2). CVD is responsible for 37% of deaths attributable to noninfectious causes of individuals under 70 years of age worldwide (3), and approximately one in four deaths in the US (4). In 2018, the prevalence of CVD excluding hypertension was 11.8% among adults over 18 years of age in the US (2). The prevalence also increases by age and was 28.5% among those age ≥ 65 years (2).

A large, multinational cohort study found that approximately 70% of CVD cases are attributable to modifiable risk factors (5). Thus, non-medical interventions that can reduce CVD and its underlying risk factors, such as management of anxiety and social isolation, exercise, and a healthy diet, are critical. There is also considerable interest in the beneficial role that companion animals play in human health, including the potentially protective effect of pet ownership on CVD. In 2020, 45 and 26% of US households had a dog or a cat, respectively (6). Thus, a positive association between pet ownership and CVD could provide a strong basis for implementation of a population-based prevention strategy.

Several hypotheses have been proposed that may explain the potential relationship between pet ownership and CVD. First, it is hypothesized that the pet ownership-CVD association is mediated by psychological and physiological changes that occur when a pet is present (7–12). For example, Allen et al. (7) found that the increase of heart rate and blood pressure while performing challenging math problems was significantly smaller among people who had their pet present than those without their pet present. In 1993, Patronek and Glickman (10) coined the term “pet preventable fraction” as the percent of myocardial infarctions and death that pet ownership could prevent. However, this fraction has not been firmly established for CVD. Second, it is hypothesized that the protective benefit of pet ownership is partly mediated by increased exercise (13, 14), particularly in the case of dog ownership. Some studies have found that dog owners have higher levels of physical activity than non-dog owners (15–18). In contrast, cat ownership has not been found to be associated with physical activity (15, 18). Thus, reduced CVD risk among pet owners may not be merely mediated by increased physical activity.

However, existing studies regarding the association between pet ownership and CVD have not yielded consistent results, showing either positive, negative, mixed, or no associations. Several meta-analyses have attempted to clarify the relationship, but their findings have also differed. For instance, one meta-analysis found modestly lower cardiovascular mortality among cat owners and pet owners compared to non-cat and non-pet owners, respectively, but the differences were not statistically significant (19). Another meta-analysis found that dog owners had a reduced risk of cardiovascular mortality compared to non-dog owners, but the study did not adjust for potential confounding factors (20). In a meta-analysis by Yeh et al. (21), pet ownership was associated with a lower adjusted cardiovascular mortality in the general population compared with CVD patients.

These discrepancies may result from several methodological differences across the studies. First, as discussed in a systematic review, the CVD risk factors considered in these studies differed (22). One of the meta-analyses excluded covariates from their analysis (20), while others included them (19, 21). Second, the studies considered populations with varying age groups. For instance, Chowdhury et al. (23) evaluated the association between pet ownership and survival among hypertensive adults between 65 and 84 years of age, while Maugeri et al. (24) evaluated the association between dog ownership and CVD among adults between 25 and 44 years. A few existing meta-analyses were also conducted by integrating results from studies with participants of various age groups. For example, Kramer et al. included a study with participants between 33 and 85 (25), a study with participants between 65 and 84 (23), and an earlier study with participants who were “adults” with unspecified age (12). El-Qushayri et al. also included studies with participants of varying age from 5 to 17 years (26) to 50–95 years (27). Yeh et al. included studies whose participants were 44.5–72.6 years.

The association between pet ownership and CVD may differ between age groups due to changes in the relationship between the pet and owner across the lifespan. Pets may be sole companions for some senior people, particularly women who are more likely to live alone than age-matched men (3). Pets may also mitigate feelings of loneliness and social isolation among senior people who live alone leading to a reduced risk of CVD.

In this study, we aim to evaluate the association between pet ownership and CVD risk while considering a comprehensive list of risk factors and confounders. We hypothesize that the effect of pet ownership on CVD risk may differ by sex and age groups. Therefore, we further evaluate potential effect modification due to age and sex, which has not been well understood in the literature. The varying effect of pet ownership across subgroups of participants may explain the inconsistent results from previous studies.

## Methods

### Study population

Our study population included 6,632 individuals who were participants of the American Gut Project (AGP) between 2012 and 2020. The AGP employed a cross-sectional study design with voluntary response sampling. Participants enrolled in the project through either the Indiegogo or FundRazr crowdsourcing websites and completed a demographics, health, and lifestyle survey. Our study was limited to participants ≥40 years of age who resided in the US and answered multiple choice survey questions about pet ownership, cardiovascular disease, and age (28) (see Supplementary Survey). The lower age limit in this study was chosen as individuals ≥40 years are significantly more likely to have age-related forms of CVD compared with those <40 years who are more likely to have hereditary forms (29).

### Ethical considerations

Participants’ consent for the AGP was obtained under Institutional Review Board human research subject protocols from University of Colorado, Boulder (Protocol 12-0582, 12/2012-03/2015) or University of California, San Diego (Protocol 141853, 02/2015-present). All data were deidentified and publicly available. Raw data were obtained using redbiom, a utility that allows for accessing and processing of publicly available data stored in Qiita (30). The protocol of this study was reviewed by the Institutional Review Board at Indiana University and was determined to be non-human subjects research (Protocol 1910657990).

### Exposure—pet ownership

The main exposures of interest were cat ownership and/or dog ownership. Cat ownership was defined as having a cat(s) and dog ownership as having a dog(s).

### Outcome—CVD

The outcome of interest was history of CVD. The answer to the survey question, “Have you ever been diagnosed with coronary artery disease, heart disease, heart attack, or stroke?” determined the history of CVD. Cases were defined as participants who were diagnosed with CVD by a medical professional. CVD controls were defined by those participants who answered “No” to this question.

### Covariates

The AGP collected a comprehensive list of covariates that may impact the association between CVD and pet ownership. Demographic factors included age group (40–64 years and ≥ 65 years), sex (female or male), and the highest level of education attained (up to some high school, high school graduate to undergraduate, some graduate school or graduate degree). We dichotomized age into two age groups using the cut point of ≥65 years as previous studies have suggested that the rate of increased CVD risk is relatively stable up to age 65, then increases significantly after age 65 (31). We conducted sensitivity analysis using alternative cut off values (i.e., 60 years) to evaluate the robustness of our analysis. Race or ethnicity was self-reported by participants as Caucasian, Asian or Pacific Islander, African American, Hispanic, or “other.” As more than 90% of participants self-identified as Caucasian, race or ethnicity was collapsed into two levels, Caucasian and non-Caucasian. Height and weight were self-reported by participants and used to calculate body mass index (BMI).

### Statistical analysis

Data analysis was conducted using RStudio, Version 4.1.4 (32). The univariate comparisons of covariates between cases and controls were conducted by Pearson’s Chi-square test and two-sample *t*-test for categorial and continuous variables, respectively (Table 1). The association between pet ownership on CVD (odds ratio, or OR) was estimated *via* multivariable-adjusted logistic regression models. Potential confounders were included as covariates in the logistic regression models.

**Table 1:** Study population characteristics.

Characteristic	All (*n* = 6,632)	CVD (*n* = 405, 6.1%)	No CVD (*n* = 6,227, 93.9%)	*p* value
Pet ownership (exposure)				
Cat ownership	1,829 (27.6%)	71 (17.5%)	1,758 (28.2%)	<0.01
Dog ownership	2,398 (36.2%)	142 (35.1%)	2,256 (36.2%)	0.61
Age (years)				<0.01
40–64	3,729 (56.2%)	182 (44.9%)	4,507 (72.4%)	
≥65	2,903 (43.8%)	223 (55.1%)	1,720 (27.6%)	
Sex				<0.01
Female	3,535 (53.3%)	136 (33.6%)	3,399 (54.6%)	
Male	3,053 (46.0%)	268 (66.2%)	2,785 (44.7%)	
Race				<0.01
Caucasian	6,013 (90.7%)	383 (94.6%)	5,630 (90.4%)	
Non-Caucasian	604 (9.1%)	22 (5.4%)	582 (9.4%)	
Sleep				<0.01
≥ 7-h	3,771 (56.9%)	203 (50.1%)	3,568 (57.3%)	
<7-h	2,844 (42.9%)	200 (49.4%)	2,644 (42.5%)	
BMI				<0.01
Underweight	175 (2.6%)	8 (2.0%)	167 (2.6%)	
Normal	3,286 (49.5%)	141 (34.8%)	3,185 (51.1%)	
Overweight	2,108 (31.8%)	178 (44.0%)	1930 (31.0%)	
Obese	1,006 (15.2%)	78 (19.3%)	928 (14.9%)	
Alcohol use				<0.01
Daily	1,109 (16.7%)	63 (15.6%)	1,046 (16.8%)	
Regularly (12–20 times/month)	1,203 (18.1%)	109 (26.9%)	1,094 (17.6%)	
Occasionally (4–11 times/month)	1,214 (18.3%)	53 (13.1%)	1,161 (18.6%)	
Rarely (1–3 times/month)	1813 (27.3%)	90 (22.2%)	1723 (27.7%)	
Never	1,303 (19.6%)	109 (26.9%)	1,194 (19.2%)	
Smoking				0.63
Smoker	241 (3.6%)	17 (4.2%)	224 (3.6%)	
Non-smoker	6,374 (96.1%)	387 (95.5%)	5,987 (96.1%)	
Exercise				<0.01
Daily	1,420 (21.4%)	90 (22.2%)	1,330 (21.4%)	
Regularly (12–20 times/month)	2,585 (39.0%)	179 (44.2%)	2,406 (38.6%)	
Occasionally (4–11 times/month)	1712 (25.8%)	75 (18.5%)	1,637 (26.3%)	
Rarely (1–3 times/month)	705 (10.6%)	42 (10.4%)	663 (10.6%)	
Never	199 (3.0%)	18 (4.4%)	181 (0.3%)	
Education				<0.01
Up to high school graduate	142 (2.1%)	14 (3.5%)	128 (2.1%)	
Associate’s to bachelor’s degree	2,367 (35.7%)	171 (42.2%)	2,196 (35.3%)	
Graduate school or graduate degree	4,101 (61.8%)	219 (54.1%)	3,882 (62.3%)	
Unspecified	22 (0.3%)	1 (0.2%)	21 (0.3%)	

We further tested the secondary hypotheses that there are interactions among cat ownership, dog ownership, age, and sex. We considered five additional logistic regression models with various combinations of interaction terms representing potential effect modification among these factors (Table 2). We first evaluated the effect of cat ownership and dog ownership on CVD risk using a multivariate logistic regression model without considering any potential interactions (i.e., Model 1). We adjusted for age, sex, race, BMI, sleep, alcohol use, smoking, exercise, and education. Model 2 included a cat-by-dog interaction term. Model 3 included an age-by-cat ownership interaction term. Model 4 included an age-by-dog ownership interaction term. Model 5 included both age-by-cat and age-by-dog ownership interaction terms. Model 6 included interaction terms for sex-by-cat ownership and sex-by-dog ownership. All models adjusted for the same covariates other than pet ownership, age, and sex. We conducted stepwise model selection using likelihood ratio tests to determine the optimal model based on the data (33). The likelihood ratio test is calculated as the ratio of the log likelihood of the simpler model relative to the more complex model, with the test statistic approximating a chi-square distribution. When comparing two models, a nested model that was more parsimonious was preferred unless an extended model significantly improved the model fit (*p* < 0.05).

**Table 2:** Logistic regression models for estimating effect of pet ownership with or without interactions.

Model 1	CVD ~ age + cat ownership + dog ownership + sex + other covariates*
Model 2	CVD ~ age + cat ownership + dog ownership + sex + other covariates* + (cat ownership x dog ownership)
Model 3	CVD ~ age + cat ownership + dog ownership + sex + other covariates* + (age x cat ownership)
Model 4	CVD ~ age + cat ownership + dog ownership + sex + other covariates* + (age x dog ownership)
Model 5	CVD ~ age + cat ownership + dog ownership + sex + other covariates* + (age x cat ownership) + (age x dog ownership)
Model 6	CVD ~ age + cat ownership + dog ownership + sex + other covariates* + (sex x cat ownership) + (sex x dog ownership)

*Other covariates include amount of sleep, BMI category, sex, race, smoking status, exercise frequency, alcohol use frequency, education.

## Results

### Participant characteristics

The participants’ characteristics and univariate comparisons between cases and controls are shown in Table 1. Our study population included 405 CVD cases (6.1%) and 6,227 controls (93.9%). Cat owners comprised 27.6% of the population and showed significantly lower proportion in cases than in controls (17.5% vs. 28.2%; *p* < 0.01). Dog owners comprised 36.2% of the population and had comparable proportions between cases and controls (35.1% vs. 36.2%, *p* = 0.61). For dichotomized covariates, a higher proportion of the population was 40–64 years (56.2%), female (53.3%), Caucasian (90.7%), sleep over 7 h/day (56.9%), and current non-smokers (96.1%). Significant differences were found between cases and controls for most of these covariates, including ≥65 years (55.1% vs. 27.6%), female (33.6% vs. 54.6%), Caucasian (94.6% vs. 90.4%), and sleep over 7 h/day (50.1% vs. 57.3%). The proportion of non-smokers did not show significant difference between cases and controls (95.5% vs. 96.1%). A few covariates (BMI, alcohol use, exercise, education) had three or more levels. The levels with the highest proportion were normal BMI (49.5%), rarely alcohol use (27.3%), exercise regularly (39.0%), and some graduate school or graduate degree (61.8%). The distributions of all these covariates were significantly different between cases and controls. For example, the cases were less likely to have normal BMI than controls (34.8% vs. 51.1%).

### The association between pet ownership and CVD

The results of the adjusted model without interaction terms are summarized in Table 3. Cat owners had significantly lower risk of CVD compared with non-cat owners (OR: 0.56 [0.42, 0.73]). There was no significant difference in the risk of CVD between dog owners and non-dog owners (*p* = 0.17). The risk of CVD was approximately 3.5 times higher among participants aged ≥65 years than among those age 40–64 (OR: 3.51 [2.80, 4.40]) and approximately 2 times higher among men than women (OR: 2.07 [1.65, 2.61]). Both obese and overweight had significantly increased risk over normal BMI, while underweight did not show any significant difference. Exercise, alcohol use and education were all significantly associated with CVD risk, while smoking was not.

**Table 3:** Estimated effects of covariates without consideration of potential interactions (i.e., Model 1).

Covariate		Odds ratio	Confidence interval (95%)	*p* value
Cat owner	Yes	0.56	0.42, 0.73	<0.01
	No	Ref	Ref	Ref
Dog owner	Yes	1.17	0.88, 1.39	0.17
	No	Ref	Ref	Ref
Age	40–64 years	Ref	Ref	Ref
	≥ 65 years	3.51	2.80, 4.40	<0.01
Race	Non-Caucasian	0.64	0.64, 0.97	0.04
	Caucasian	Ref	Ref	Ref
Sex	Male	2.07	1.65, 2.61	<0.01
	Female	Ref	Ref	Ref
BMI (kg/m^2^)	Obese	1.95	1.41, 2.66	<0.01
	Overweight	2.07	1.61, 2.66	<0.01
	Normal	Ref	Ref	Ref
	Underweight	1.22	0.54, 2.43	0.60
Sleep	<7-h	1.45	1.16, 1.81	<0.01
	≥ 7-h	Ref	Ref	Ref
Smoking status	Smoker	1.32	0.75, 2.19	0.31
	Non-smoker	Ref	Ref	Ref
Exercise frequency	Never	0.83	0.46, 1.42	0.52
	Rarely (1–3 times/month)	0.81	0.55, 1.17	0.28
	Occasionally (4–11 times/month)	0.49	0.31, 0.66	<0.01
	Regularly (12–20 times/month)	Ref	Ref	Ref
	Daily	0.93	0.70, 1.23	0.63
Alcohol use frequency	Never	Ref	Ref	Ref
	Rarely (1–3 times/month)	0.55	0.40, 0.74	<0.01
	Occasionally (4–11 times/month)	0.45	0.31, 0.65	<0.01
	Regularly (12–20 times/month)	0.77	0.55, 1.06	0.10
Highest education attained	Daily	0.41	0.29, 0.59	0.01
	Up to high school graduate	1.50	0.77, 2.71	0.21
	Associate’s to bachelor’s degree	1.50	1.20, 1.88	<0.01
	Some graduate school or graduate degree	Ref	Ref	Ref

### Interactions between pet ownership and age or sex

Results of the likelihood ratio tests are shown in Table 4. Model 5 performed significantly better than the other models and was selected as the final model. We followed the principle of parsimony during model selection, wherein an extended model is only selected if it fits the data significantly better than a reduced model (*p* < 0.05). Sex-by-pet ownership interactions did not improve the data fitting over the non-interaction model (Model 6 vs. Model 1) so were not considered further in additional models. The results of Model 5, which included interaction terms for age-by-cat ownership and age-by-dog ownership, are shown in Table 5. Adjusted ORs, 95% confidence intervals, and value of *p*s for the final model are shown in Table 5. As a result of the interactions, each participant falls into one of eight groups depending on their level of cat ownership, dog ownership, and age group. Each of these eight groups has a different CVD risk (Table 5).Participants who were 40–64 years, non-cat owners, and non-dog owners were considered the reference group. Compared to the reference group, all other groups had a significantly different risk of CVD.

**Table 4:** Results of likelihood ratio tests for model selection.

Models compared (reduced vs. extended)	Likelihood ratio test	Value of *p*	df
1 vs. 2	0.49	0.48	1
1 vs. 3	5.62	0.02	1
1 vs. 4	6.91	<0.01	1
3 vs. 5	6.94	<0.01	1
4 vs. 5	5.65	0.02	1
1 vs. 5	12.56	<0.01	2
1 vs. 6	2.98	0.23	2

Likelihood ratio test = chi-square test statistic for the likelihood-ratio test: −2(LogL_nested model_ – LogL_complex model_). df = degrees of freedom for the χ ^2^ test statistic defined as the number of covariances fitted for the two models.

**Table 5:** Estimated effects of covariates with pet ownership-by-age interactions (i.e. Model 5).

Covariate		Odds ratio	Confidence interval (95%)	Prob(>|*z*|) value of *p*
Age × pet ownership interaction	≥ 65 years, cat owner, not dog owner (*N* = 306)	2.97	1.98, 4.47	<0.01
	40–64 years, cat owner, not dog owner (*N* = 876)	0.40	0.26, 0.61	<0.01
	≥ 65 years, cat owner, and dog owner (*N* = 109)	2.42	1.50, 3.91	<0.01
	40–64 years, not cat owner, not dog owner (*N* = 1,850)	Ref	Ref	Ref
	≥ 65 years, not cat owner, and dog owner (*N* = 326)	3.14	2.09, 4.72	<0.01
	40–64 years, not cat owner, and dog owner (*N* = 1,425)	1.53	1.12, 2.08	0.01
	≥ 65 years, not cat owner, and not dog owner (*N* = 1,202)	3.85	2.84, 5.24	<0.01
	40–64 years, cat owner, and dog owner (*N* = 538)	0.60	0.35, 1.03	0.03
Sleep	<7 h	1.43	1.15, 1.78	<0.01
	≥ 7 h	Ref	Ref	Ref
BMI (kg/m^2^)	Obese	1.90	1.39, 2.59	<0.01
	Overweight	2.03	1.58, 2.62	<0.01
	Normal	Ref	Ref	Ref
	Underweight	1.21	0.53, 2.40	0.62
Sex	Male	2.08	1.65, 2.62	<0.01
	Female	Ref	Ref	Ref
Race	Non-Caucasian	0.64	0.40, 0.97	0.04
	Caucasian	Ref	Ref	Ref
Smoking status	Smoker	1.37	0.78, 2.29	0.25
	Non-smoker	Ref	Ref	Ref
Exercise frequency	Never	0.88	0.49, 1.50	0.65
	Rarely (1–3 times/month)	0.83	0.56, 1.19	0.32
	Occasionally (4–11 times/month)	0.46	0.32, 0.66	<0.01
	Regularly (12–20 times/month)	Ref	Ref	Ref
	Daily	0.96	0.72, 1.26	0.75
Alcohol use frequency	Never	Ref	Ref	Ref
	Rarely (1–3 times/month)	0.54	0.40, 0.74	<0.01
	Occasionally (4–11 times/month)	0.46	0.32, 0.66	<0.01
	Regularly (12–20 times/month)	0.76	0.55, 1.05	0.10
	Daily	0.41	0.28, 0.59	<0.01
Highest education attained	Up to high school graduate	1.51	0.77, 2.73	0.20
	Associate’s to bachelor’s degree	1.49	1.19, 1.87	<0.01
	Some graduate school or graduate degree	Ref	Ref	Ref

Participants who were 40–64 years who owned a cat had lower risk of CVD than age-matched participants without a cat, regardless of whether they also owned a dog. Cat-owning Participants who were 40–64 years who did not own a dog had the lowest risk of CVD (OR: 0.40 [0.26, 0.61]) compared to the reference group. Participants aged 40–64 years who owned both a cat and dog had the second lowest risk (OR: 0.60 [0.35, 1.03]) compared to the reference group.

**Figure 1:**
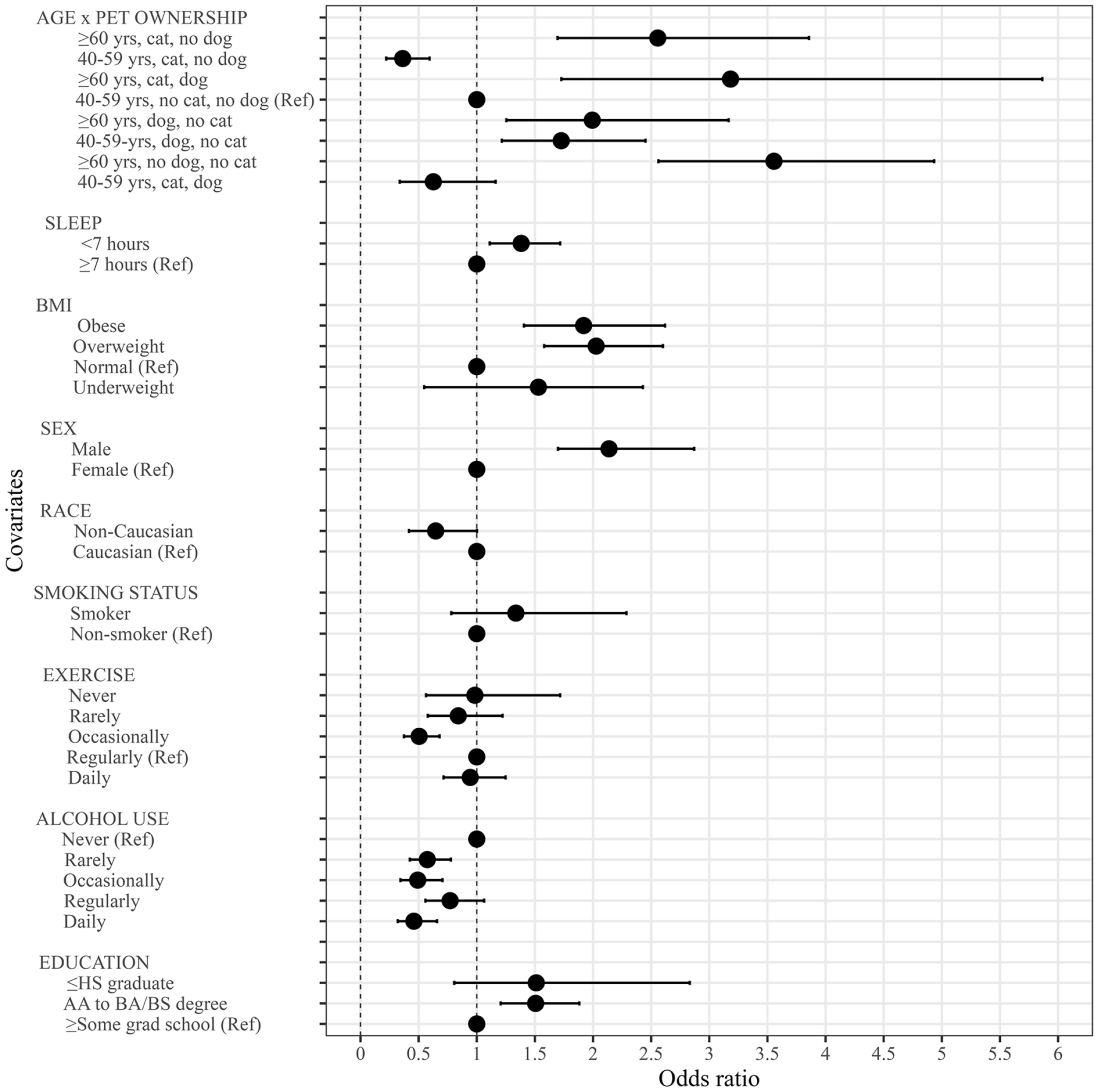
Association between covariates and CVD risk based on the final model (i.e., Model 5).

For participants ≥65 years, those who owned both a cat and dog had the lowest CVD risk (OR: 2.42 [1.50, 3.91]). On the other hand, participants ≥65 years who did not own a cat or a dog had the highest risk (OR: 3.85 [2.84, 5.24]). The other participants ≥65 years had varying levels of CVD risk based on the profiles of cat and dog ownership with odds ratios ranging from 2.97 to 3.85. The risk of CVD for all age and pet ownership groups was significantly different from the reference group at *p* < 0.05 significance level. The risks of CVD for these groups are summarized in Table 5 and depicted in Figure 1.

**Figure 2:**
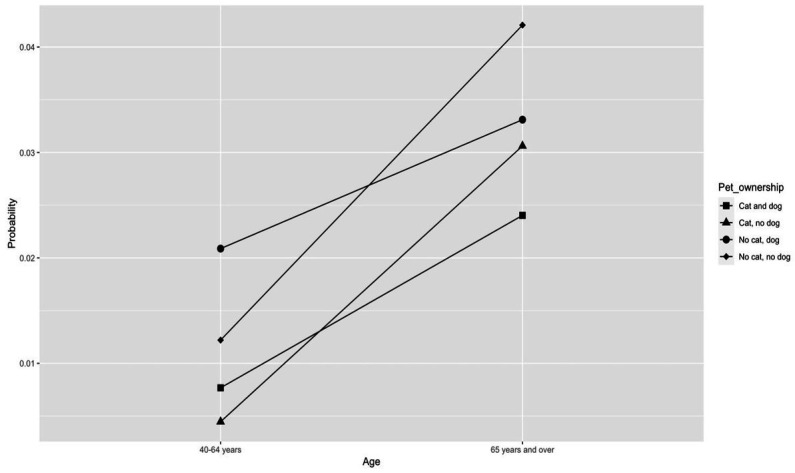
Estimated probability of CVD as a function of pet ownership and age, assuming an individual with a fixed covariate profile (i.e., >7 h sleep, normal BMI, female, white, non-smoker, regular exerciser, rare drinker, and some grad school; i.e. the most common people in our study).

To illustrate this relationship due to the complex interactions, we further described the effect modification of age on pet ownership in Figure 2. In this figure, we estimated participants’ risk of CVD for the most common covariate profile of participants in the study. For all participants, the estimated risk of CVD increases with age, but at a varying rate by the pet ownership status. For ≥65-year participants, owning no pets was associated with the highest risk of CVD. In contrast, for 40-64-year participants, owning a dog but no cat was associated with the highest CVD risk. Owning both a cat and a dog was associated with the lowest CVD risk among participants ≥65 years, while owning a cat but no dog was associated with the lowest risk among those 40–64 years.

We further repeated all analyses using an alternative cut-off value for age groups (i.e., 40–59 years and ≥60 years) as a sensitivity analysis. There were no appreciable difference from these results; thus, the conclusion remains the same.

## Discussion

We found a significant association between pet ownership and CVD risk in our study population. In addition, we found that age group was an effect modifier of this association. For those ≥65 years, owning both a cat and dog was associated with the lowest risk of CVD, while owning no cat or dog was associated with the highest risk of CVD. Further, for those 40–64 years, the lowest risk of CVD was associated with having a cat but no dog, while those with a dog but no cat had the highest CVD risk. A participant’s sex did not significantly modify the associations between pet ownership and CVD. While some prior studies have shown that owning a pet may be associated with reduced risk of CVD, others showed no association. Our findings may help explain the discrepancies in prior studies. Our results suggest that it could be especially beneficial for people ≥65 years to have both cat and dog with respect to cardiovascular health. However, for people aged 40–64 years, having a cat may have sufficient benefit, while having a dog will increase rather than decrease the risk of CVD.

Owning both a cat and dog may be associated with the lowest risk of CVD among ≥65-year participants due to reduced feelings of loneliness and social isolation. This is consistent with existing literature (34). Feelings of loneliness are lowest in middle-aged adults and highest in late adulthood (35). For adults 65–84 years, one study found that feelings of loneliness or social isolation were significantly lower among current or past dog owners than never dog owners (36). In a study of adults ≥60 years, pet owners were 36% less likely to report a feeling of loneliness compared with non-pet owners (37).

Our findings also suggest that owning a dog may increase the risk of CVD among those who are 40–64 years and cat owners. One potential reason is that dog ownership adds responsibilities to life, such as providing food, water, and exercise and managing veterinary care. While cat owners also provide daily food and water, they may not spend time as much time exercising their animals. Cat owners also visit the veterinarian less frequently than dog owners. A 2016 American Veterinary Medical Association survey found 54% of cat owners and 83% of dog owners visited the veterinarian at least once in 2016 (38). Adding these at a time in life when many people are already busy with work and family demands may be a source of stress. Further, dog owners aged 40–64 years may tend to own breeds that confer more stress or other demands.

Another reason the association between pet ownership and cardiovascular disease might differ depending on age may be that cardiovascular reactivity tends to be higher in older than younger individuals. For example, blood vessel responses to stress are impaired as people age due to vascular aging (39). Several experimental studies have found an association between pet ownership and cardiovascular parameters among adults, especially blood pressure and heart rate (7, 40, 41). Maintaining a healthy blood pressure can reduce the rate of vascular aging (42, 44). As pet ownership was associated with blood pressure reductions in several studies, pets may help reduce the rate of vascular aging in people as they age.

Our findings should be viewed considering a few limitations. For example, future studies may benefit from adjusting for pet breed and for pet care demands, such as time spent feeding or walking pets. In addition, further investigation of the association between pet ownership and the adult gut microbiome, contrasting people with and without CVD, may also elucidate the mechanism by which pet ownership is associated with CVD.

Another limitation is that the cases and controls were significantly different, as shown in Table 1. Compared to cases, controls tended to be younger, female and Caucasian, in the normal BMI category, more likely to have attended graduate school and be occasional exercisers, and more likely to be regular alcohol users. While these variables were controlled covariates in the logistic regression models, it is possible that there could be unmeasured confounding of some of the variables, such as BMI. It is possible that residual differences between cases and controls could confound results. It is also likely that the imbalance with respect to other unmeasured factors may contribute to the observed association. Future studies could resolve this with propensity scores.

A shortcoming of this dataset is that, while there was a race and ethnicity question on the questionnaire, the response encoders ultimately classified participants by race (i.e., Caucasian, Asian or Pacific Islander, African American, Hispanic, or “other”). Thus, information about ethnicity was missing in this analysis.

There are also public health measures that could be implemented based on these findings. In particular, the benefit of pet ownership to the cardiovascular health of those ≥65 years suggests that people of this age group should not give up their pets, including people who reside in retirement or assisted living centers. However, not all retirement or assisted living centers accept pets. In 2019, approximately 75% of for-profit retirement living residences accepted pets and the proportion of non-profit residences accepting pets is much lower (43). This is contrary to the US Department of Housing and Urban Development requirement that all properties designated for “elderly or handicapped persons” may not discriminate against individuals with a “common household pet” (45).

Finally, as this study is cross-sectional, a cause-and-effect relationship between pet ownership, some covariates (exercise, sleep, alcohol use, and exercise frequency), and CVD cannot be drawn. Reverse causation can not be ruled out with this study as the order of events is unknown. For instance, a participant may have been advised to obtain a pet after being diagnosed with CVD. However, the finding of effect modification of the pet ownership by age by CVD relationship suggests that future prospective studies, or propensity score analyses, where cause and effect may more easily be interpreted, are indicated.

## Data availability statement

Publicly available datasets were analyzed in this study. This data can be found at: https://www.ebi.ac.uk/ena/browser/view/PRJEB11419.

## Author contributions

KW conceived and designed the study, performed the statistical analysis, and wrote the first manuscript draft. ML and KK provided study design guidance. ML contributed to the statistical analysis. TS edited the final manuscript and assisted with data visualization. All authors contributed to the manuscript revisions, read, and approved the submitted version.

## Funding

Research funding to KW and ML has been provided by the Human Animal Bond Research Institute, Grant ID# HAB20-010. ML is supported by the National Heart, Lung and Blood Institute under award number K01HL140333.

## Conflict of interest

The authors declare that the research was conducted in the absence of any commercial or financial relationships that could be construed as a potential conflict of interest.

## Publisher’s note

All claims expressed in this article are solely those of the authors and do not necessarily represent those of their affiliated organizations, or those of the publisher, the editors and the reviewers. Any product that may be evaluated in this article, or claim that may be made by its manufacturer, is not guaranteed or endorsed by the publisher.
