# Supplementary material for: Age modifies the association between pet ownership and cardiovascular disease
Source: Front Vet Sci. 2023 May 12;10:1168629. doi: 10.3389/fvets.2023.1168629 (PMC10213240; doi:10.3389/fvets.2023.1168629)
Supplement: Supplementary file 1 [file Data_Sheet_1.PDF]

Supplementary Survey

GENERAL QUESTIONNAIRE

1.) What is your race/ethnicity?

a. Caucasian

b. Asian or Pacific Islander

c. African American

d. Hispanic

e. Other** *Please explain in the supplemental answer section*

2.) Do you have a dog(s)?

a. Yes** *Please explain in the supplemental answer section*

b. No

3.) Do you have a cat(s)?

a. Yes** *Please explain in the supplemental answer section*

b. No

4.) Do you have other pet(s)at home:

a. Yes** *Please explain in the supplemental answer section*

b. No

5.) Which is your dominant hand?

a. I am right-handed

b. I am left-handed

c. I am ambidextrous

6.) What is your highest level of education?

a. Did not complete high school

b. High School or GED equivalent

c. Some college or technical school

d. Associate's degree

e. Bachelor's degree

f. Some graduate school or professional

g. Graduate or Professional degree

7.) Have you ever been diagnosed with coronary artery disease, heart disease, heart attack, stroke?

a. I do not have this condition

b. Diagnosed by a medical professional (doctor, physician assistant)

c. Diagnosed by an alternative medicine practitioner

d. Self-diagnosed
